# Impacts of urea and 3,4-dimethylpyrazole phosphate on nitrification, targeted ammonia oxidizers, non-targeted nitrite oxidizers, and bacteria in two contrasting soils

**DOI:** 10.3389/fmicb.2022.952967

**Published:** 2022-07-28

**Authors:** Qing Wang, Ziting Zhao, Manyao Yuan, Zhijun Zhang, Shanshuai Chen, Yunze Ruan, Qiong Huang

**Affiliations:** ^1^College of Tropical Crops, Hainan University, Haikou, China; ^2^Sanya Nanfan Research Institute of Hainan University, Sanya, China; ^3^Institute of Agricultural Environment and Soil, Hainan Academy of Agricultural Sciences, Haikou, China

**Keywords:** neutral soil, acidic soil, ammonia oxidizers, nitrite oxidizers (NOB), 3,4-dimethylpyrazole phosphate (DMPP)

## Abstract

This study explored the effects of combined urea and 3,4-dimethylpyrazole phosphate (DMPP) on several components critical to the soil system: net nitrification rates; communities of targeted ammonia oxidizers [ammonia-oxidizing archaea (AOA) and bacteria (AOB) and complete ammonia-oxidizing bacteria (comammox)]; non-targeted nitrite-oxidizing bacteria (NOB) and bacteria. We conducted the study in two contrasting soils (acidic and neutral) over the course of 28 days. Our results indicated that DMPP had higher inhibitory efficacy in the acidic soil (30.7%) compared to the neutral soil (12.1%). The abundance of AOB and *Nitrospira*-like NOB were positively associated with nitrate content in acidic soil. In neutral soil, these communities were joined by the abundance of AOA and *Nitrobacter*-like NOB in being positively associated with nitrate content. By blocking the growth of AOB in acidic soil—and the growth of both AOB and comammox in neutral soil—DMPP supported higher rates of AOA growth. Amplicon sequencing of the 16S rRNA gene revealed that urea and urea + DMPP treatments significantly increased the diversity indices of bacteria, including Chao 1, ACE, Shannon, and Simpson in the acidic soil but did not do so in the neutral soil. However, both urea and urea + DMPP treatments obviously altered the community structure of bacteria in both soils relative to the control treatment. This experiment comprehensively analyzed the effects of urea and nitrification inhibitor on functional guilds involved in the nitrification process and non-targeted bacteria, not just focus on targeted ammonia oxidizers.

## Introduction

Nitrification is a microbially driven process that converts ammonia (NH_3_) *via* nitrite (NO_2_^–^) to nitrate (NO_3_^–^). The process is mainly controlled by autotrophic organisms that are able to grow with NH_3_ and/or NO_2_^–^ as their unique energy source. Autotrophic nitrification occurs soil in one of two ways: in a traditional two-step process by ammonia-oxidizing bacteria (AOB), archaea (AOA), and nitrite-oxidizing bacteria (NOB) (Stein and Klotz, 2016); or in a single organism by recently discovered complete ammonia-oxidizers (comammox) within the *Nitrospira* genus (Daims et al., 2015; Van Kessel et al., 2015). Past studies focusing on soil nitrifiers have mostly concentrated on canonical ammonia oxidizers (AOB and AOA), as ammonia oxidation is the rate-limiting step in nitrification (Prosser and Nicol, 2012), and ignored the key role of NOB in soil nitrification. Nitrification is a crucial process of the nitrogen (N) cycle, impacting N substrate availability for plant and microorganisms, NO_3_^–^ leaching, and nitrous oxide (N_2_O) emissions (Gruber and Galloway, 2008). Considering that ammonium-based fertilizer consumption will continue to increase (Liu et al., 2015), it is important to develop effective management practices that reduce nitrification rates and improve N use efficiency for plants, hopefully mitigating environmental damage.

Nitrification inhibitors (NIs) applied in combination with N-based fertilizers have been considered an effective method to mitigate the adverse effects of nitrification on both the environment and N use efficiency (Shi et al., 2016b; Li et al., 2020). 3,4-Dimethylpyrazole phosphate (DMPP) is a highly effective and commonly used commercial nitrification inhibitor in the agricultural systems (Zerulla et al., 2001; Irigoyen et al., 2003). However, the inhibition efficiency of DMPP on nitrification is reported to differ significantly across soil types (Li et al., 2019; Yin et al., 2021). Environmental factors such as temperature (Nair et al., 2021), moisture (Menéndez et al., 2012), and soil properties such as pH and soil organic matter in different types of soils significantly affect the efficacy of DMPP (Shi et al., 2016a,b). These factors mostly influence the bio-availability of DMPP and the activity or function of key ammonia oxidizers, which then affects the inhibition efficiency of DMPP on nitrification. For example, the temperature can have significant effects on DMPP mobility and its degradation rate in soils (Irigoyen et al., 2003). Soil pH affects the niche differentiation of canonical ammonia oxidizers; AOB is dominant in neutral/alkaline soils with high ammonium-N input (Shen et al., 2008; Jia and Conrad, 2009), and AOA is dominant in low ammonia acidic soils (Zhang et al., 2012). High organic matter content in soils may adsorb DMPP and decrease its inhibitory efficiency on nitrification (Shi et al., 2016a). Therefore, the inhibitory effects of DMPP on nitrification might be closely related to its inhibition on functionally active ammonia oxidizers in different types of soils, but few studies have explored this concept.

Ammonia-oxidizing bacteria, AOA, and comammox coexist in terrestrial ecosystems and compete for the substrate NH_3_ (Prosser et al., 2020). Many studies about the impacts of DMPP on ammonia oxidizers are unclear, probably due to their unknown effects on nitrifying communities (Shi et al., 2016a; Fan et al., 2019). For example, a recent study showed that DMPP specifically and effectively inhibited AOB growth, while having no effect on AOA in three types of soils (Yin et al., 2021). Others reported that DMPP obviously reduced the abundance and activity of both AOA and AOB (Florio et al., 2014; Bachtsevani et al., 2021). In addition, recent studies suggest that DMPP significantly inhibited the growth of comammox in various types of soils (Li et al., 2019, 2020). Previous studies reported that AOA or AOB proliferation accelerated when AOB or AOA growth was selectively inhibited. However, little is known about the contribution of comammox to nitrification in various types of soils (Hink et al., 2018; Fan et al., 2019; Zhao et al., 2020; Yin et al., 2021). Thus, the potentially important roles of AOA, AOB, and comammox on DMPP-induced inhibition of nitrification remain unclear.

The effects of DMPP on non-targeted microbes like NOB—which are fed with substrates acquired from ammonia oxidation—as well as total bacterial communities remain unknown. Nitrite oxidation is found to be more sensitive than ammonia oxidation in artificially disturbed soils, implying that NOB plays a key role in nitrification (Gelfand and Yakir, 2008; Roux-Michollet et al., 2008). Canonical ammonia oxidizers provide substrates for NOB by converting NH_3_ into NO_2_^–^. Some NOBs, such as the *Nitrospira* group, can also convert urea into NH_3_ and CO_2_, providing the substrate NH_3_ for ammonia oxidizers without urease genes, thus forming a “reciprocal feeding relationship” between canonical ammonia oxidizers and nitrite oxidizers (Daims et al., 2016). DMPP application reduces NO_2_^–^ concentration by inhibiting the activity of ammonia oxidizers and might indirectly affect the growth of NOB. Very few studies have explored the effects of DMPP on non-targeted bacterial communities and the results are contradictory. For example, Luchibia et al. (2020) revealed that DMPP application did not alter the bacterial community composition based on 16S rRNA gene sequencing in a grassland soil. However, Bachtsevani et al. (2021) found that DMPP had significant effects on bacterial community composition in two different types of cropland soils. To fully understand the impacts of DMPP on soil biological functions, it is necessary to explore its non-targeted effects on soil microbial communities, including NOB and bacteria—not just on targeted ammonia oxidizers.

The purpose of this experiment is to study the impacts of DMPP and urea on soil nitrification rate, communities of ammonia oxidizers, non-target nitrite oxidizer, and total bacteria in two contrasting soils. Based on these data, we assessed the interactions between ammonia oxidizers and NOB using the functional genes abundances. We hypothesized that (1) urea might vary in its impact on soil nitrification and functional gene abundance based on soil type; (2) DMPP would have lower inhibitory efficacy on nitrification in neutral soil than in acidic soil due to the higher organic matter concentration of the former; (3) DMPP inhibits nitrification by targeting canonical ammonia oxidizers and comammox—depending on soil type—but has no significant effects on non-target NOB and bacteria.

## Material and methods

### Experimental site and soil sampling

In July 2020, we collected two types of arable soils from different sites: an acidic soil (Maize, pH 4.74)from Qiyang, Hunan province, South China (26°42′N, 110°35′ E); and a neutral soil (Maize, pH 6.95) from Changchun, Jilin province, North China (45°15′ N, 124°18′ E). Both soils are widely distributed and are from major grain-producing regions in China. The acidic soil from Qiyang county is classified as paleudults, while the neutral soil from Jilin is classified as Mollisol according to the FAO soil classification system (FAO, 2015). At each sampling site, five topsoil samples (0–20 cm deep) were mixed into a composite sample. Field moisture soils were sieved (<2 mm) and saved under 4°C for soil microcosm incubation within a week. Soils were air-dried for chemical analysis.

### Soil chemical analyses

All soil parameters were analyzed according to the method of Lu (2000). Soil pH was measured with the ratio of 1:2.5 (w/v, soil/water) using a pH meter (Mettler Toledo, Switzerland). Soil NH_4_^+^ and NO_3_^–^ contents were extracted in a ratio of 1:5 with 2M KCl and measured by a continuous flow analyzer (Skalar + system, Netherlands). Organic matter (OM) was measured by using K_2_Cr_2_O_7_ oxidation method. Total nitrogen (TN) was determined after digestion of the sample by using 5 ml of concentrated H_2_SO_4_ and a semi-automatic Kjeldahl nitrogen analyzer. Available phosphorus (AP) was determinated in soils adopts ammonium fluoride extraction method and molybdemum-antimony colorimetric method. Soil available potassium (AK) was determined by extraction with ammonium acetate. Basic soil properties are shown in Supplementary Table 1.

### Soil microcosm experiment

Soils were pre-incubated with a water holding capacity (WHC) of 40% at 25°C for one week to stimulate the activity of microorganisms. When preparing for the formal incubation, we placed 20 g (dry weight) of the soil samples in 120 ml plastic bottles. We conducted a three-treatment microcosm experiment composed of nil-treated control (CK); single application of urea (Urea); and urea plus DMPP (Urea + DMPP). All Urea treatments received the final concentration of 100 mg N kg^–1^ soil, and the Urea + DMPP treatment received DMPP at a rate of 1.5% urea-N (i.e., the commercial rate). The added solutions (or deionized water for the CK) resulted in soil at 60% of WHC. All small plastic bottles were sealed by parafilm and then incubated at 25 °C in the dark for 28 days. All treatments were performed in triplicate. During the incubation process, we added sterile water to the surface of the soil sample every 3–4 days to replenish the water lost. Three replicate soils in each treatment were destructively collected on the day of setup (0 day), 7, 14, 28 days after incubation, 5 g of soil were taken to measure pH value, and 5 g were used to assess soil NH_4_^+^-N and NO_3_^–^-N content. Residual soil samples (10g) were frozen at −80°C for DNA extraction.

The calculation of the net nitrification rates (n) are based on the formula proposed by Persson and Wirén (1995), and the specific calculation is as follows:


(1) n(d-0d)7=[(NO-3-N)-d7(NO-3-N)]d0/7

(2) n(d-0d)14=[NO-3-N)-d14(NO-3-N)]d0/14

(3) n(d-0d)28=[NO-3-N)-d28(NO-3-N)]d0/28

where (NO_3_^–^-N) _*d*0_, (NO_3_^–^-N)_*d*7_, (NO_3_^–^-N)_*d*14_, (NO_3_^–^-N)_*d*28_ are NO_3_^–^-N contents in the soil on days 0, 7, 14, and 28, respectively (Table 1).

**TABLE 1:** Net nitrification rates under different treatments in acidic and neutral soil during incubation.

Soil types	Time intervals	Net nitrification rate (mg NO_3_^–^-N kg^–1^ soil day^–1^)			Percent Inhibition by DMPP (%)
		CK	Urea	Urea + DMPP	
Acidic soil	d_0_-d_7_	0.45 ± 0.03b	0.70 ± 0.06a	0.50 ± 0.02b	28.6
	d_0_-d_14_	0.68 ± 0.02b	0.81 ± 0.01a	0.66 ± 0.01b	18.5
	d_0_-d_28_	0.40 ± 0.01c	0.88 ± 0.01a	0.61 ± 0.02b	30.7
Neutral soil	d_0_-d_7_	1.36 ± 0.06e	7.31 ± 0.03a	5.52 ± 0.13b	24.5
	d_0_-d_14_	1.07 ± 0.01d	6.39 ± 0.63a	5.96 ± 0.13b	6.7
	d_0_-d_28_	0.90 ± 0.02d	3.54 ± 0.03a	3.11 ± 0.03c	12.1

Different letters represent significant differences under different treatments (*P* < 0.05).

### DNA extraction and quantitative PCR

Soil genomic DNA was extracted from 0.5 g of soil samples on days 0 and 28 with the RNeasy Powersoil DNA Elution kit (Qiagen, Germany). The extracted DNA concentration and purity were evaluated using Qubit4.0 Fuorometer (Invitrogen, United States).

The abundances of the key nitrifying including ammonia oxidizers and nitrite oxidizers were quantified using the ABI 7500 system (ABI, United States). Target genes, primers, and thermocycling conditions are shown in Supplementary Table 2. The total PCR reaction volume was 20 μL, which contains 10 μL 2 × SYBR Premix Ex Taq (Takara, China), 0.5 μL 50 × Rox Reference Dye (Takara, China), 0.4 μL forward and reverse primers, 2 μL template DNA (10–20 ng) and 7.2 μL RNase-free water. PCR products of targeted genes (AOA, AOB, Comammox *amoA* and *Nitrobacter*-like NOB *nxrA* and *Nitrospira*-like NOB *nxrB*) were inserted into PMD18-T plasmids. Standard curves were constructed using 10-fold serial dilutions of plasmids DNA from one representative clone containing the correct targeted gene. Melting curve analysis was conducted between 65 and 95°C at the end of amplification to evaluated the specificity of PCR products. The amplification efficiency of the five functional genes ranged between 85 and 93%, with the R^2^ values ≥ 0.99.

### Illumina miseq sequencing of 16S rRNA gene and bioinformatic analysis

We also studied the impacts of urea with DMPP on diversity and community composition of non-targeted bacteria. The V4-V5 region of the 16S rRNA gene in soils on day 0 and 28 was amplified with the primer pairs 515F (GTGCCAGCMGCCGCGGTAA) and 907R (CCGTCAATTCCTTTGAGTTT) (Stubner, 2002). A unique barcode was adapted to the 5′end to distinguish different samples. Each 25 μL PCR reaction mixture included 12.5 μL 2 × Premix Taq, 0.5 μL forward and reverse primers, 2 μL template DNA (10–20 ng) and 9 μL RNase-free water. The PCR amplification conditions involved 3 min at 94°C; 28 cycles of 94°C for 30 s; 56°C for 30 s; and 72°C for 30 s. The final stage of this lasted for 6 min at 72°C.

PCR products of 16S rRNA gene were used with the Illumina Miseq platform at Majorbio, Shanghai, China. The raw sequence data were demultiplexed and filtered using QIIME quality filters. The reads were truncated at any position with > 3 consecutive quality scores ≤ 25. Sequences ≤ 200 bp were discarded before further analysis. Chimeras sequences were detected using a *de novo* algorithm. The trimmed sequences were clustered into operational taxonomic units (OTUs) at a 97% similarity cutoff using the Usearch, and the representative sequences were selected to annotate taxonomic information against the SILVA database using the RDP classifier with an 80% confidence threshold. All raw sequences can be found at NCBI under accession number PRJNA839188 and sample accession numbers SAMN28513062-SAMN28513073 for acidic soil and SAMN28513074-SAMN28513085 for neutral soil (Supplementary Table 3).

### Statistical analysis

We used one-way analysis of variance (ANOVA) in SPSS Statistics 20 to assess the treatment effects on net nitrification rate, the abundances of ammonia oxidizers and nitrite oxidizers. Spearman’s correlation was conducted to evaluate the associations between AOB, AOA, comammox, *Nitrobacter*-like NOB or *Nitrospira*-like NOB abundances and NO_3_^–^-N contents. Differences of *P* < 0.05 were considered statistically significant. Principal coordinates analysis (PCoA) was used to assess the β-diversity of bacterial community composition between various treatments using the “vegan” package for R (Version 3.1.2). Permutational multivariate analysis of variance (PERMANOVA) was performed to test whether the differences in bacterial community composition across treatments were significant.

## Results

### Soil pH

**FIGURE 1:**
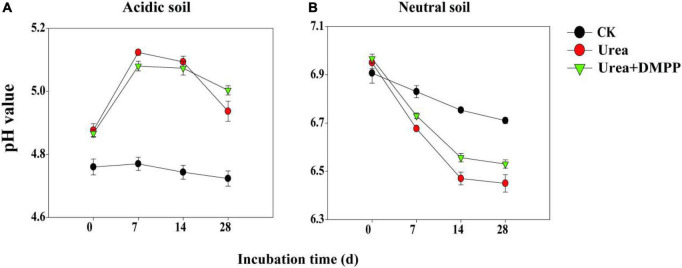
Dynamics of soil pH under different treatments during incubation of panel **(A)** acidic soil and **(B)** neutral soil. Bars indicate standard error, *n* = 3.

In the acidic soil, pH in the CK treatment did not change significantly during the incubation period, ranging from 4.74 on day 0 to 4.72 on day 28 (Figure 1A). Soil pH reached its peak in the first week of the urea and urea + DMPP treatments and then decreased subsequently, though the pH remained much higher than it was throughout the entire incubation period in the CK treatment. At the end of incubation, soil pH in the urea treatment was slightly lower than that in urea + DMPP treatment.

In the neutral soil, soil pH among all treatments dropped throughout incubation, though different treatments have distinct effects on pH (Figure 1B). In the CK treatment, soil pH dropped regularly from 6.91 on day 0 to 6.71 on day 28. Both the urea and urea + DMPP treatments decreased the pH, but the urea treatment did so slightly more aggressively. For example, soil pH in the urea treatment declined from 6.95 on day 0 to 6.45 on day 28, while in the urea + DMPP treatment, pH decreased from 6.96 to 6.53. After the 28-day incubation, soil pH was lower in the urea and urea + DMPP treatments than it was in the CK treatment.

### Dynamics of exchangeable NH_4_^+^-N and NO_3_^–^-N concentrations and net nitrification rates

**FIGURE 2:**
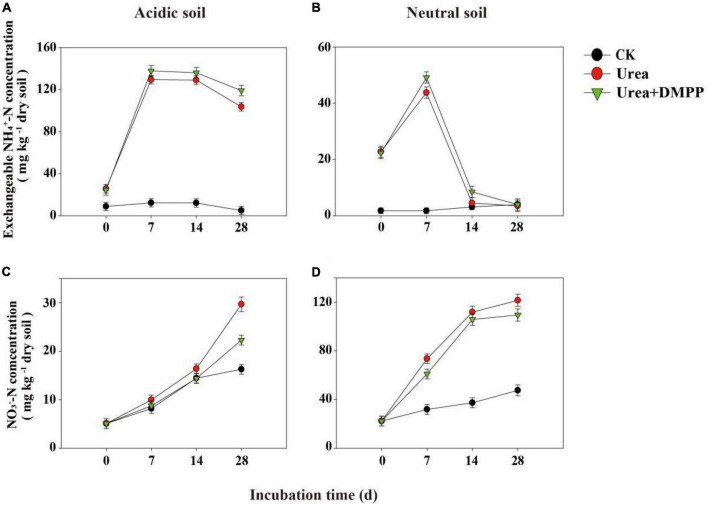
Exchangeable NH_4_^+^-N, and NO_3_^–^-N concentrations under different treatments during incubation in panels **(A,C)** acidic soil and **(B,D)** neutral soil. Bars indicate standard error, *n* = 3.

In both soils, the exchangeable NH_4_^+^-N concentration of CK-treated soils remained at a low level and changed little during the 28-day incubation (Figures 2A,B). In acidic soil, urea significantly increased the concentration of exchangeable NH_4_^+^-N, peaking 134.74 mg/kg in the first week (Figure 2A). After the first week, exchangeable NH_4_^+^-N regularly decreased. DMPP significantly prevented the reduction of exchangeable NH_4_^+^-N during the whole incubation period.

In neutral soil, urea rapidly enhanced the exchangeable NH_4_^+^-N concentration in the first week, though on day 14 the concentration of NH_4_^+^-N declined quickly (Figure 2B). Urea + DMPP reduced the concentration of exchangeable NH_4_^+^-N to the level of the CK treatment after 28 days, although DMPP slowed the rate of NH_4_^+^-N decline.

In the acidic and neutral soils, the NO_3_^–^-N concentrations in the CK treatment increased over time, indicating a stable nitrification activity occurred in soils (Figures 2C,D). Urea amendment significantly stimulated NO_3_^–^-N production during the incubation period and had higher net nitrification rates compared to the CK treatment, while DMPP inhibited NO_3_^–^-N production (Figures 2C,D). In the acidic soil, the net nitrification rate was < 1 mg kg^–1^ soil day^–1^ (Table 1). In the CK treatment, the average net nitrification rate was 0.40 mg NO_3_^–^-N kg^–1^ soil day^–1^ during the whole incubation period. Urea significantly increased the net nitrification rates to 0.70 mg NO_3_^–^-N kg^–1^ soil day^–1^ in the first week and 0.88 mg NO_3_^–^-N kg^–1^ soil day^–1^ during the 28-day incubation period. However, DMPP significantly decreased the average net nitrification rates by 28.6% (0.50 mg NO_3_^–^-N kg^–1^ soil day^–1^) in the first week and 30.7% (0.61 mg NO_3_^–^-N kg^–1^ soil day^–1^) during the 28-day incubation period compared to the urea treatment. In the neutral soil, the average net nitrification rate throughout the incubation period in the CK treatment was 0.90 mg NO_3_^–^-N kg^–1^ soil day^–1^. Urea strongly enhanced the net nitrification rates to 7.31 mg NO_3_^–^-N kg^–1^ soil day^–1^ in the first week and 3.54 mg NO_3_^–^-N kg^–1^ soil day^–1^ during the 28-day incubation period. Compared to the urea treatment, DMPP decreased the average net nitrification rates by 24.5% (5.52 mg NO_3_^–^-N kg^–1^ soil day^–1^) in the first week and 12.1% (3.11 mg NO_3_^–^-N kg^–1^ soil day^–1^) during the 28-day incubation period.

### Abundances of ammonia oxidizers and nitrite oxidizers

**FIGURE 3:**
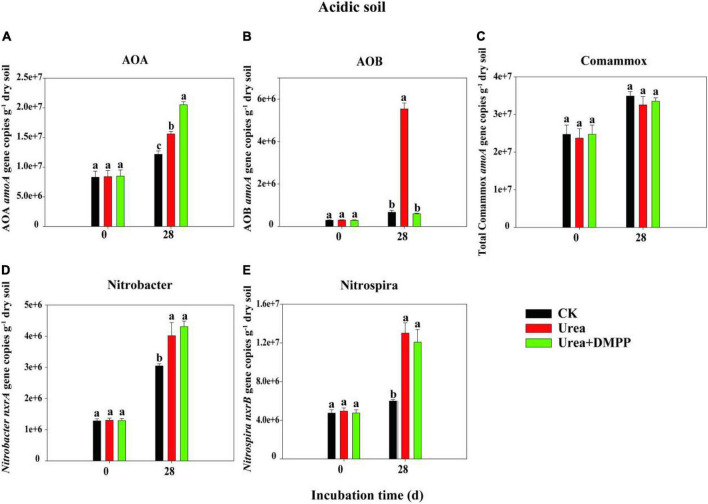
Dynamics of panel **(A)** AOA; **(B)** AOB; **(C)** total comammox *amoA* gene; **(D)** *Nitrobacter*-like *nxrA*; and **(E)** *Nitrospira*-like *nxrB* gene copies in acidic soil. Bars indicate standard errors, *n* = 3. Different letters represent significant differences under different treatments (*P* < 0.05).

**FIGURE 4:**
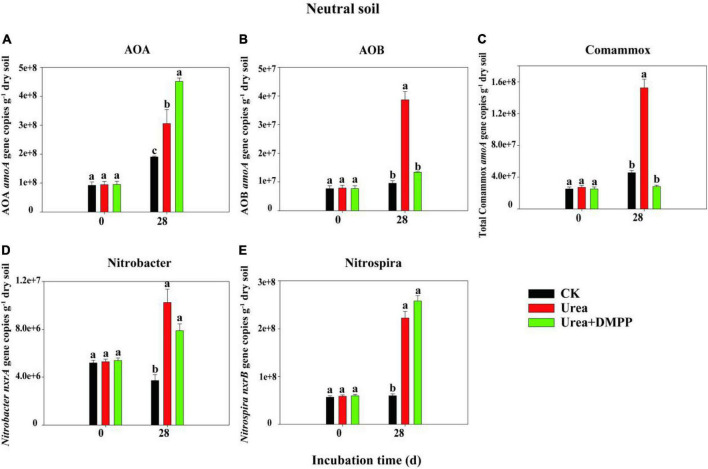
Dynamics of panel **(A)** AOA; **(B)** AOB; **(C)** total comammox *amoA* gene; **(D)** *Nitrobacter*-like *nxrA*; and **(E)** *Nitrospira*-like *nxrB* gene copies in neutral soil. Bars indicate standard errors, *n* = 3. Different letters represent significant differences under different treatments (*P* < 0.05).

The changes in the abundances of nitrifiers in acidic (Figure 3) and neutral soil (Figure 4) were quantified using qPCR analysis. The abundance of AOA across all treatments ranged from 8.29 × 10^6^ to 1.95 × 10^7^ copies g^–1^ soil in the acidic soil and from 9.21 × 10^7^ to 4.51 × 10^8^ copies g^–1^ soil in the neutral soil. Compared to the CK treatment, urea significantly increased AOA abundance on day 28 by 60.54% in acidic soil (Figure 3A) and by 31.63% in neutral soil (Figure 4A). Unexpectedly, compared to the urea treatment, urea + DMPP treatment significantly enhanced AOA abundance on day 28 by 31.63% in the acidic soil and by 32.28% in the neutral soil. The abundance of AOB across all treatments ranged from 2.95 × 10^5^ to 5.54 × 10^6^ copies g^–1^ soil in the acidic soil (Figure 3B) and 7.68 × 10^6^ to 3.87 × 10^7^ copies g^–1^ soil in the neutral soil (Figure 4B). Urea treatment led to an obvious increase in AOB abundance on day 28 compared to the CK treatment in both soils, and DMPP significantly decreased AOB abundance. The abundance of comammox across all treatments ranged from 2.47 × 10^7^ to 3.49 × 10^7^ copies g^–1^ soil in the acidic soil (Figure 3C) and 2.52 × 10^7^ to 1.52 × 10^8^ copies g^–1^ soil in the neutral soil (Figure 4C). Urea showed no significant effect on comammox abundance in the acidic soil, though urea significantly increased comammox in the neutral soil. DMPP did not influence comammox abundance in the acidic soil, but significantly decreased it in the neutral soil.

The abundance of the *Nitrobacter*-like NOB across all treatments varied from 1.28 × 10^6^ to 4.31 × 10^7^ copies g^–1^ soil in the acidic soil (Figure 3D) and 3.71 × 10^6^ to 1.02 × 10^7^ copies g^–1^ soil in the neutral soil (Figure 4D). The abundance of the *Nitrospira*-like NOB across all treatments varied from 4.74 × 10^6^ to 1.30 × 10^7^g^–1^ in the acidic soil (Figure 3E) and 5.65 × 10^7^ to 2.58 × 10^8^ copies g^–1^ soil in the neutral soil (Figure 4E). In both soils, compared to the CK treatment, urea significantly increased *Nitrobacter*- and *Nitrospira*-like NOB abundances (*P* < 0.05). However, DMPP had no impact on them compared to urea-only treatment (*P* > 0.05).

**FIGURE 5:**
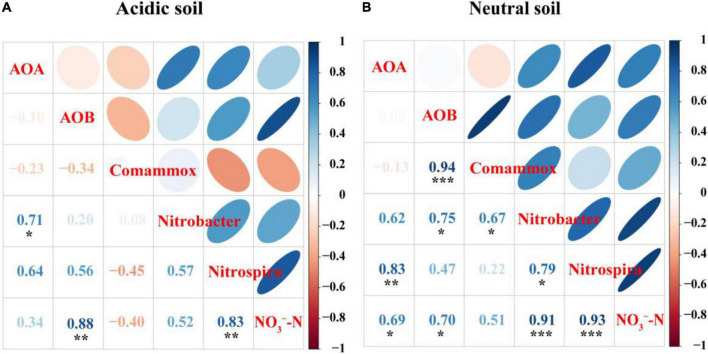
Spearman’s correlation analyses of AOA, AOB, comammox, and *Nitrobacter*-like NOB and *Nitrospira*-like NOB abundances and NO_3_^–^-N concentration in panel **(A)** acidic soil and **(B)** neutral soil.

Spearman’s correlation analysis suggested that the abundances of AOB (R^2^ = 0.88, *P* < 0.01), and *Nitrospira*-like NOB (R^2^ = 0.83, *P* < 0.01) were significantly correlated with NO_3_^–^-N concentration in acidic soil (Figure 5A). In neutral soil, the abundances of AOA (R^2^ = 0.70, *P* < 0.05), AOB (R^2^ = 0.69, *P* < 0.05), *Nitrobacter*-like NOB (R^2^ = 0.91, *P* < 0.001), and *Nitrospira*-like NOB (R^2^ = 0.93, *P* < 0.001) were significantly correlated with NO_3_^–^-N concentration (Figure 5B).

### Bacterial α-diversity and community composition

After rarefaction including quality trimming and chimera removal, a total of 1,32,7750 high-quality sequences (45,000–65,000 sequences per sample) were obtained for the 24 samples based 16S rRNA gene amplicon sets. Sequences obtained *via* Miseq sequencing were used to assess the changes in soil bacterial diversity and community composition. Urea significantly increased α-diversity indices of bacteria including Chao 1, ACE, Shannon, and Simpson in acidic soil while having no bacterial diversity in the neutral soil. In contrast, DMPP showed no significant effects on α-diversity of bacteria in either soil compared with urea-only treatment (Table 2).

**TABLE 2:** Bacterial α-diversity under different treatments.

Soil types	Treatments	Chao1	Ace	Shannon	Simpson
Acidic soil	CK	1247.99 ± 41.93b	1257.55 ± 42.69b	5.75 ± 0.04b	0.988 ± 0.01c
	Urea	1565.77 ± 22.51a	1574.15 ± 21.04a	6.14 ± 0.02a	0.993 ± 0.02a
	Urea + DMPP	1493.44 ± 51.6a	1502.66 ± 53.26a	6.03 ± 0.04a	0.992 ± 0.01a
Neutral soil	CK	1853.39 ± 14.19ab	1895.56 ± 33.1a	6.58 ± 0.03ab	0.996 ± 0.01a
	Urea	1827.37 ± 10.09b	1836.68 ± 10.17a	6.53 ± 0.02b	0.996 ± 0.02a
	Urea + DMPP	1902.25 ± 30.05a	1843.48 ± 4.68a	6.63 ± 0.01a	0.996 ± 0.03a

Different letters present significant differences under different treatments (*P* < 0.05).

**FIGURE 6:**
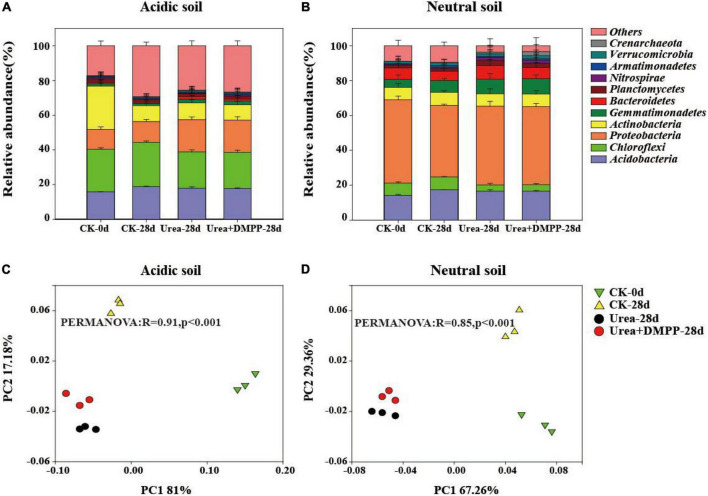
Relative abundance of the main phyla (with abundance > 1% of total sequences) across different treatments in the acidic soil **(A)** and neutral soil **(B)**. PCoA and PERMANOVA was conducted to assess the dissimilarity in bacterial community structure across the treatments in the acidic soil **(C)** and neutral soil **(D)**.

Both soils possessed a similar bacterial community composition at the phyla (Figure 6) and genus (Supplementary Figure 1) levels. In the acidic soil, the dominant phyla contained *Acidobacteria* (15.82–18.89%), *Chloroflexi* (20.76–25.46%), *Proteobacteira* (11.40–18.76%), *Actinobacteira* (8.90–25.13%), *Gemmatimonadetes* (1.26–2.16%), *Bacteriodetes* (0.50–1.34%), *Planctomycetes* (1.72–2.20%), and *Nitrospirae* (0.47–0.71%) (Figure 6A). Urea and urea + DMPP treatments significantly decreased the relative abundance of *Acidobacteria* and *Chloroflexi*, while strongly enhancing the relative abundances of *Proteobacteira*, *Gemmatimonadetes*, and *Bacteriodetes*, compared to the CK.

The main genus were *Sinomonas* (5.81–6.02%), *Streptomyces* (4.15–5.59%), *Arthrobacter* (2.37–2.58%), *Kaistobacter* (1.86–6.68%), *Burkholderia* (1.53–2.50%), and *Candidatus_Koribacter* (1.19–2.48%) (Supplementary Figure 1A). Urea and urea + DMPP treatments significantly increased the relative abundance of genus *Kaistobacter*, but decreased the relative abundance of *Burkholderia*, *Sinomonas*, *Streptomyces*, *Arthrobacter* and *Candidatus _ koribacter*.

In the neutral soil, the dominant phyla were *Acidobacteria* (14.25–17.75%), *Chloroflexi* (3.53–7.86%), *Proteobacteira* (41.03–47.93%), *Actinobacteira* (7.10–7.48%), *Gemmatimonadetes* (4.64–8.69%), *Bacteriodetes* (5.46–7.85%), *Planctomycetes* (1.44–3.03%), *Nitrospirae* (0.63–1.59%), and *Verrucomicrobia* (1.29–1.92%) (Figure 6B). Urea significantly decreased the relative abundance of *Acidobacteria* and *Chloroflexi*, while strongly increasing the relative abundances of *Proteobacteira*, *Gemmatimonadetes*, *Bacteriodetes*, and *Planctomycetes*. DMPP, however, had no effect on bacterial relative abundance compared to the urea treatment.

The main genus were *Kaistobacter* (12.31–16.34%), *Cupriavidus* (1.10–8.29%), *Flavisolibacter* (1.24–2.56%), *Rhodoplanes* (1.12–1.91%), *Candidatus _ Nitrosophaera* (1.34–1.60%), *Massilia* (1.08–1.47%), *Thermomonas* (1.10–1.44%), and *Methylibium* (1.03–1.15%) (Supplementary Figure 1B). Compared with CK, urea amendment significantly increased the relative abundance of *Rhodoplanes*, but decreased the relative abundance of *Kaistobacter*, *Flavisolibacter*, *Cupriavidus*, *Massilia*, and *Thermomonas*. Compared with urea treatment, urea + DMPP treatment significantly increased the relative abundance of *Candidatus_Nitrososphaera* and *Methylibium*, while significantly decreased the relative abundance of *Kaistobacter* and *Flavisolibacter*.

We used PCoA and PERMANOVA to study how bacterial community structure varied among the different treatments in both soils (Figures 6C,D). The first two components in PCoA explained 98.18 and 96.62% of the variability in acidic (Figure 6C) and neutral soils (Figure 6D), respectively. These findings were confirmed by the PERMONOVA analysis (R = 0.91, *P* < 0.001; R = 0.85, *P* < 0.001). Compared to the CK treatment, urea significantly altered the bacterial community structure in both soils. However, compared to the urea-only treatment, DMPP did not significantly alter bacterial community structure.

## Discussion

We analyzed the effects of urea and urea with DMPP on nitrification in two contrasting soils. DMPP was more effective at impeding net nitrification rates in the acidic soil than in the neutral soil (Table 2), which was inconsistent with some previous studies (Shi et al., 2016a,b) but supports a more recent study (Bachtsevani et al., 2021). One possible explanation is that DMPP, as a type of heterocylic compound, can be adsorbed onto soil organic matter (McCarty and Bremner, 1989), thus diminishing the efficacy of DMPP hindering nitrification (Barth et al., 2008). This study showed that more organic matter resided in the neutral soil than the acidic soil, potentially reducing DMPP efficiency in neutral soil. Similarly, the adsorption of other nitrification inhibitors, such as nitrapyrin, onto soil organic matter affects the inhibitory potential of the substance (Fisk et al., 2015). Thus, the effect of DMPP on nitrification strongly depended on soil type and corresponding soil properties.

The concentration of substrate NH_3_, which is an energy source for autotrophic ammonia oxidizers, affected the niche differentiation of AOA, AOB and comammox (Prosser and Nicol, 2012; Hu and He, 2017). Previous studies showed that AOA had a higher affinity for NH_3_ than AOB (Hu and He, 2017). Thus, many studies have reported that AOA preferentially grows in acidic soils with low NH_3_ availability (Zhang et al., 2012), while AOB prefers neutral or alkaline soils with high inorganic NH_4_^+^ input (Jia and Conrad, 2009). Previous research observed either no change or a reduction in the abundance of AOA in response to high NH_4_^+^-N in agricultural soils (Wang et al., 2016; Sun et al., 2021). However, this paper showed that urea significantly stimulated the growth of AOA in both acidic and neutral soils with high NH_4_^+^ input. Furthermore, Lehtovirta-Morley et al. (2016) reported that a new AOA strain, *Nitrosocosmicus franklandianus*, was isolated from a agricultural soil and could adapt to high NH_3_ conditions. Jung et al. (2022) also found that terrestrial AOA might not have substantially higher affinities to ammonia than AOB. These findings suggests that AOA have an affinity for a wide range of NH_3_ concentrations.

There are few studies on the impacts of NH_3_ concentration on the abundance of comammox because it was only recently discovered (Daims et al., 2015). *Nitrospira inopinata*, the only pure strain of comammox, has a higher NH_3_ affinity than most AOA strains and all known AOB strains, and lives an oligotrophic lifestyle (Kits et al., 2017). However, in this study, urea showed no significant effects on comammox abundance in acidic soil, but significantly stimulated its abundance in neutral soil. We speculate that the differences in comammox communities in different types of soils might lead to distinct responses to substrate NH_3_ addition; some comammox taxon might not be strictly limited to oligotrophic environments (Li et al., 2019; Wang et al., 2019; Sun et al., 2021). Further physiological studies that isolate AOA, AOB, and comammox strains are necessary to evaluate NH_3_-driven niche differentiation among these ammonia oxidizers.

We observed an inhibition of AOB growth in acidic soil and inhibition of both AOB and comammox growth in neutral soil, but we did not observe an inhibition of AOA growth by DMPP, which is consistent with previous studies (Shi et al., 2016a; Fan et al., 2019; Li et al., 2020). Most commonly used nitrification inhibitors such as DCD, nitrapyrin, and DMPP are believed to act as metal chelators, binding copper to the active site of the *amo*B subunit (Ruser and Schulz, 2015). DMPP appears ineffective on AOA, possibly due to a structural difference in the *amo*B subunit of these ammonia oxidizers (Tolar et al., 2017). Our results also revealed that the population size of AOA was stimulated under high ammonia environment when its competitor (like AOB or comammox) was repressed in both soils, which is supported by recent studies by Hink et al. (2018) and Fan et al. (2019). Beeckman et al. (2018) reported that comammox might present a similar ammonia oxidation mechanism as AOB, explaining why AOB and comammox responded similarly to DMPP. We observed a close association between the abundance of AOB and comammox (R^2^ = 0.94, *P* < 0.001). However, previous most studies did not take the activity of comammox into account. This study reveals for the first time that AOA growth accelerates when the activities of both AOB and comammox are inhibited in neutral soil. Thus, the nitrification-inhibition mechanism of DMPP might strongly depends on soil type.

*Nitrobacter*-like NOB are dominant in high N concentrations and possess reduced substrate affinity compared to *Nitrospira*-like NOB (Nowka et al., 2015). Previous studies in grassland and cropland soils reported that N fertilization stimulated the growth of *Nitrobacter*-like NOB, but had no effect on or inhibited the growth of *Nitrospira*-like NOB (Ma et al., 2016; Han et al., 2018; Ouyang and Norton, 2020). In our study, the population size of *Nitrobacter*- and *Nitrospira*-like NOB grew with the addition of urea in both soils, a finding with concurs with recent studies in paddy and agricultural soils (Kong et al., 2019; Sun et al., 2021). We conclude that the impacts of N fertilization on *Nitrobacter*- and *Nitrospira*-like NOB are closely associated with soil type.

The influences of DMPP on nitrite oxidizers are still unclear. A recent study reported that DMPP inhibited the growth of *Nitrobacter*-like NOB but not *Nitrospira*-like NOB in acidic and alkaline soils at a high dose rate (Bachtsevani et al., 2021). The inhibitory effects of DMPP at the recommended dose rate on the growth of *Nitrobacter*- and *Nitrospira*-like NOB in the tested soils are not significant, probably because there is no target for DMPP in NOB. Theoretically, DMPP can only indirectly affect the growth of NOB by inhibiting ammonia oxidation process, but we did not observe this effect. In addition, a recent publication shows that some *Nitrospira*-like NOB may oxidize hydrogen under natural conditions and not necessarily rely on nitrite oxidation (Leung et al., 2022). Thus, the effect of DMPP on NOB is needed to comprehensively explore in the future due to their high community and functional diversity.

We investigated the potential influences of short-term urea and DMPP on the diversity and community composition of non-targeted bacteria in two contrasting soils. Short-term urea increased the α-diversity indices of bacteria in the acidic soil, but did not affect the indices in neutral soil. This suggests that the effects of short-term urea on bacterial α-diversity depends on soil type. We found that DMPP had no significant effects on the α-diversity indices of bacteria when compared to the urea-only treatment, which concurs with a recent study (Bachtsevani et al., 2021). Our results did, however, show that urea significantly altered the bacterial community structure—the relative abundance of bacterial main phyla increased or decreased in both soils, which contradicts a previous study (Luchibia et al., 2020). *Acidobacteria* and *Chloroflexi* are oligotrophic groups that have slow growth rates (Fierer et al., 2007); thus urea significantly decreased their relative abundances. *Proteobacteria*, *Bacteroidetes*, and *Planctomycetes* are copiotrophic bacteria that favor nutrient-rich environments (Fierer et al., 2012), and they are stimulated by urea when compared to the CK treatment. However, we did not discern a notable change when DMPP was added to the urea.

## Conclusion

This study comprehensively evaluated the impacts of DMPP on ammonia oxidizers and non-targeted NOB and bacteria in two contrasting soils. Our results showed that urea greatly stimulated net nitrification rates in neutral soil more so than in acidic soil, whereas DMPP had a reduced inhibitory effect on nitrification in the neutral soil than it did in the acidic soil. DMPP did not affect AOA and NOB abundance in either soil, but did strongly inhibit AOB growth in the acidic soil, and inhibited both AOB and comammox *Nitrospira* growth in the neutral soil. In both soils, DMPP was accompanied by decreased net nitrification rates. Short-term urea and urea with DMPP only significantly increased bacterial α-diversity in acidic soil, but altered the community structure in both soils. Our study enhances the understanding of the effects of urea and nitrification inhibitors on targeted ammonia oxidizers as well as on non-targeted NOB and bacteria in different types of soils. Future works that explore specific nitrification inhibitors with molecular ecology methods are needed to assess the relative roles of ammonia oxidizers and NOB in the nitrification of diverse agroecosystems.

## Data availability statement

The datasets presented in this study can be found in online repositories. The names of the repository/repositories and accession number(s) can be found in the article/Supplementary material.

## Author contributions

QW contributed to the design of the research and revised the manuscript. ZiZ conducted the experiment and participated in drafting the manuscript. MY, SC, ZhZ, and YR contributed to analyze the results. All authors have read and approved the final manuscript.
