# Supplementary material for: Impacts of urea and 3,4-dimethylpyrazole phosphate on nitrification, targeted ammonia oxidizers, non-targeted nitrite oxidizers, and bacteria in two contrasting soils
Source: Front Microbiol. 2022 Jul 28;13:952967. doi: 10.3389/fmicb.2022.952967 (PMC9374486; doi:10.3389/fmicb.2022.952967)
Supplement: Supplementary file 1 [file Data_Sheet_1.docx]

**Supplementary Information**

**Impacts of urea and 3,4-dimethylpyrazole phosphate (DMPP) on nitrification, targeted ammonia oxidizers, non-targeted nitrite oxidizers, and bacteria in two contrasting soils**

**Qing Wang^1,2^, Ziting Zhao^1,2^, Manyao Yuan^1^, Shanshuai Chen^1,2^, Zhijun Zhang^3^, Yunze Ruan^1,2^, Qiong Huang^1,2*^**

^1^College of Tropical Crops, Hainan University, Haikou, Hainan 570228, China

^2^Sanya Nanfan Research Institute of Hainan University, Hainan University, Sanya, Hainan 572025, China

^3^Institute of Agricultural Environment and Soil, Hainan Academy of Agricultural Sciences, Haikou, Hainan 571100, China

*Corresponding author: Qiong Huang Email:994351@hainanu.edu.cn

**Table S1** Chemical properties of the studied soil samples.

| Samples | pH | Organic matter (OM) (g kg^-1^) | Total N (TN) (g kg^-1^) | Available phosphorus (AP) (mg kg^-1^) | Available potassium (AK) (mg kg^-1^) | Exchangeable NH_4_^+^-N (mg kg^-1^) | NO_3_^-^-N (mg kg^-1^) |
| --- | --- | --- | --- | --- | --- | --- | --- |
| Acidic soil | 4.74 | 10.10 | 0.87 | 49.18 | 105.55 | 33.07 | 3.36 |
| Neutral soil | 6.95 | 25.01 | 1.01 | 176.4 | 114.75 | 21.54 | 11.78 |

**Table S2** Quantitative PCR primer sets and amplification conditions used in this study.

| **Target gene** | **Primer name** | **Primer sequence (5’-3’)** | **Quantitative PCR Amplification conditions** | **Reference** |
| --- | --- | --- | --- | --- |
| AOA *amoA* | Arch-amo23F | ATGGTCTGGCTWAGACG | 95 °C for 5 min, 40 cycles of 94°C for 30 s, 53 °C for 30 s and 72 °C for 1 min | Tourna et al., 2008 |
|  | Arch-amoA616R | GCCATCCATCTGTATGTCCA |  |  |
| AOB *amoA* | amoA-1F | GGGGTTTCTACTGGTGGT | 95°C for 5 min, 35 cycles of 94 °C for 20 s, 58 °C for 30 s and 72 °C for 1 min | Rotthauwe et al., 1997 |
|  | amoA-2R | CCCCTCKGSAAAGCCTTCTTC |  |  |
| Comammox | Ntsp-amoA 162F | 6-FAM-GGATTTCTGGNTSGATTGGA | 94 °C 5 min, 40 cycles of 94°C for 30 s, 58 °C for 45 s and 72 °C for 1 min | Fowler et al.,  2018 |
|  | Ntsp-amoA 359R | WAGTTNGACCACCASTACCA |  |  |
| *Nitrobacter*-like NOB | F1norA | CAGACCGACGTGTGCGAAAG | 95°C for 10 min, 40 cycles of 94°C for 20 s, 55°C for 30 s and 72°C for 1 min | Attard et al., 2010 |
|  | R2norA | TCCACAAGGAACGGAAGGTC |  |  |
| *Nitrospira*-like NOB | nxrB169f | TAC ATG TGG TGG AACA | 95°C for 10 min, 40 cycles of 94°C for 20 s, 56°C for 30 s and 72°C for 1 min | Pester et al., 2014 |
|  | nxrB638r | CGG TTC TGG TCR ATCA |  |  |

| **Table S3**. The sample accession numbers of acidic and neutral soils. | | | |
| --- | --- | --- | --- |
| Soil types | Treatment | Sample | Accession numbers |
| Acidic soil | CK-0d | C1 | SAMN28513062 |
|  |  | C2 | SAMN28513063 |
|  |  | C3 | SAMN28513064 |
|  | CK-28d | C4 | SAMN28513065 |
|  |  | C5 | SAMN28513066 |
|  |  | C6 | SAMN28513067 |
|  | Urea-28d | C13 | SAMN28513068 |
|  |  | C14 | SAMN28513069 |
|  |  | C15 | SAMN28513070 |
|  | Urea+DMPP-28d | C16 | SAMN28513071 |
|  |  | C17 | SAMN28513072 |
|  |  | C18 | SAMN28513073 |
| Neutral soil | CK-0d | C19 | SAMN28513074 |
|  |  | C20 | SAMN28513075 |
|  |  | C21 | SAMN28513076 |
|  | CK-28d | C22 | SAMN28513077 |
|  |  | C23 | SAMN28513078 |
|  |  | C24 | SAMN28513079 |
|  | Urea-28d | C31 | SAMN28513080 |
|  |  | C32 | SAMN28513081 |
|  |  | C33 | SAMN28513082 |
|  | Urea+DMPP-28d | C34 | SAMN28513083 |
|  |  | C35 | SAMN28513084 |
|  |  | C36 | SAMN28513085 |

**Fig. S1** Relative abundance of dominant bacterial genus in the acidic and neutral soils. Bacterial genus with >1% relative abundance are included.


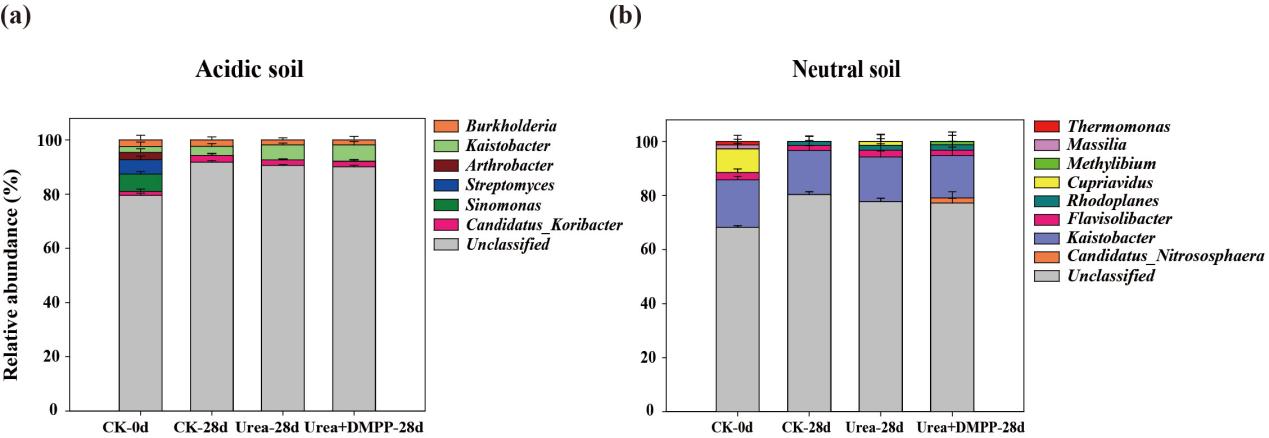


**References**

Attard E, Poly F, Commeaux C, Laurent F, Terada A, Smets BF, Recous S, Le Roux X (2010) Shifts between *Nitrospira*- and *Nitrobacter*-like nitrite oxidizers underlie the response of soil potential nitrite oxidation to changes in tillage practices. Environ Microbiol 12:315-326.  <https://doi.org/10.1111/j.1462-2920.2009.02070.x>

Fowler SJ, Palomo A, Dechesne A, Mines PD, Smets BF (2018) Comammox Nitrospira are abundant ammonia oxidizers in diverse groundwater-fed rapid sand filter communities. Environ Microbiol 20:1002–1015.

https://doi.org/10.1111/1462-2920.14033

Pester M, Maixer F, Berry D, Rattel T, Koch H, Lucker S, Nowka B, Richter A, Spieck E, Lebedeva E, Loy A, Wagner M, Daims H (2014) *Nxr*B encoding the beta subunit of nitrite oxidoreductase as functional and phylogenetic marker for nitrite-oxidizing *Nitrospira*. Environ Microbiol 16:3055–3071.  <https://doi.org/10.1111/1462-2920.12300>.

Rotthauwe JH, Witzel KP, Liesack W (1997) The ammonia monooxygenase structural gene *amoA* as a functional marker: molecular fine-scale analysis of natural ammonia-oxidizing populations. Appl Environ Microbiol 63:4704–4712. <https://doi.org/10.1128/aem.63.12.4704-4712.1997>

Tourna M, Freitag TE, Nicol GW, Prosser JI (2008) Growth, activity and temperature responses of ammonia-oxidizing archaea and bacteria in soil microcosms. Environ Microbiol 10,1357–1364. https://doi.org/10.1111/j.1462-2920.2007.01563.x
